# Branch Retinal Vein Occlusion as a Rare Presentation of Antisynthetase Syndrome

**DOI:** 10.7759/cureus.12924

**Published:** 2021-01-26

**Authors:** Swati Parida, Konstantinos T Tsaousis, Sundeep Deol, Vasilios Diakonis, Vasileios Konidaris

**Affiliations:** 1 Ophthalmology, University Hospitals of Leicester, Leicester, GBR; 2 Ophthalmology, Volos General Hospital, Volos, GRC; 3 Ophthalmology, Largo Medical Center, Tampa, USA; 4 Ophthalmology, Leicester Royal Infirmary, Leicester, GBR

**Keywords:** anti-synthetase syndrome, branch retinal vein occlusion, anti-vegf

## Abstract

A 52-year-old woman developed branch retinal vein occlusion (BRVO) in her right eye, resulting in blurred vision with visual acuities of 6/9 and 6/6-2 in the affected and unaffected eye respectively (Snellen). The patient was successfully treated with a course of eight intravitreal aflibercept injections, improving binocular visual acuity to 6/6. During the course of her ocular management, she was admitted for acute dyspnoea secondary to interstitial lung disease (ILD). The patient was diagnosed with the antisynthetase syndrome (ASS), testing positive for PL-7 auto-antibodies. ASS may have a systemic association with BRVO; although ASS is a rare condition, it should be suspected and investigated in patients with risk factors, particularly if they present with symptoms of ILD. Early ocular intervention is associated with excellent visual outcomes, and prompt diagnosis and treatment of ASS may potentially reduce risks of further retinal vaso-occlusive episodes.

## Introduction

Antisynthetase syndrome (ASS) is a rare chronic autoimmune disease, which is recognised as an important cause of autoimmune inflammatory myopathy [1], traditionally associated with dermatomyositis (DM) and polymyositis (PM). ASS is characterised by the presence of autoantibodies that target aminoacyl transfer RNA (tRNA) synthetases; 10 antisynthetase antibodies (ASAb) have been identified to date (anti-Jo-1, anti-PL-7 and anti-PL-12, anti-EJ, anti-OJ, anti-KS, anti-Zo, Anti-SC, Anti-JS and Anti-YRS), with anti-Jo-1 recognised as the most common one [2]. Although the exact prevalence of the condition is unknown, Targoff has estimated the prevalence to be 3-4/100,000 people, given that 25% of the PM/DM patients have ASAb [3]. It is twice more common in females than males and the average age at symptom onset is 55 years [4].

ASS has a diverse phenotypic spectrum, with at least one of the following conditions present in patients: interstitial lung disease (ILD), myositis, inflammatory arthritis, fever, Raynaud’s phenomenon/syndrome, and “mechanics’ hands” (painful, thickened and cracked skin on hands). While clinical features overlap with those of PM/DM, ASS has a higher incidence and severity of ILD, a higher incidence of Raynaud’s phenomenon and GORD, and it less commonly manifests with predominant myositis [2]. ILD is often severe and rapidly progressive, and the mortality and morbidity related to ASS are attributed to irreversible damage of lung parenchyma [5].

Even though ocular manifestations of dry eye, bilateral limbal follicles and retinal vasculitis in ASS patients have been previously described in the literature, this is the first known case of branch retinal vein occlusion (BRVO) in a patient with ASS [6-9]. In this report, we describe our experience and discuss learning points drawn from the management of this patient.

## Case presentation

**Figure 1:**
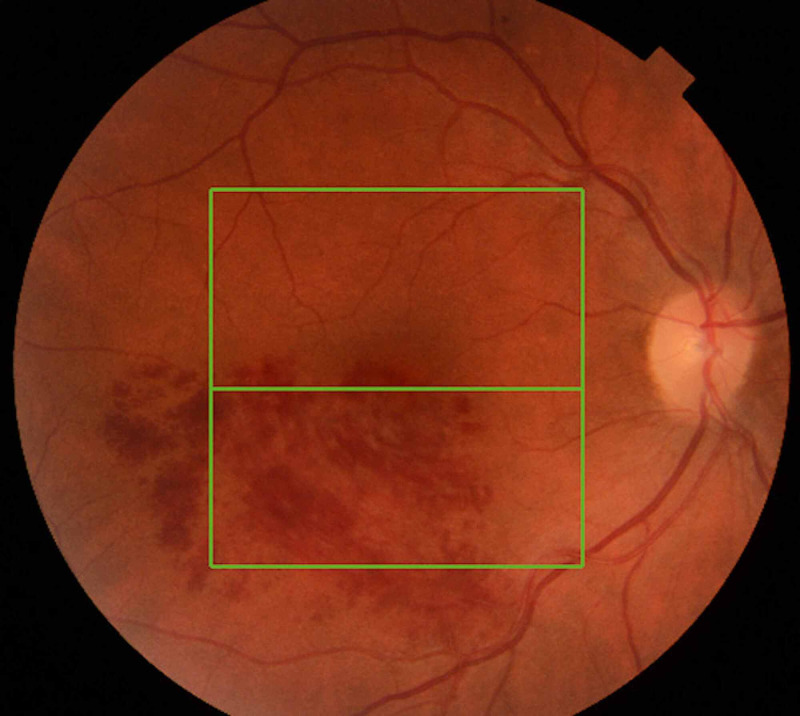
Fundus photo of the right eye at presentation with a typical image of branch retinal vein occlusion

**Figure 2:**
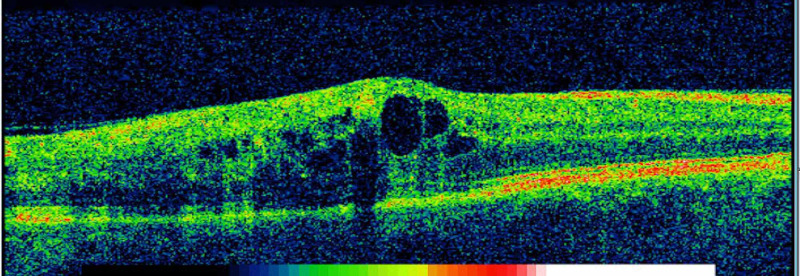
Optical coherence tomography photo of the right eye at presentation The image shows significant cystoid macular edema causing reduction of vision

A fit and healthy 52-year-old Caucasian female was referred to the Eye Casualty by her GP with a one-week history of blurred vision. At presentation, the patient’s visual acuity was 6/9 in the affected right eye and 6/6-2 in the unaffected left eye. Dilated fundoscopy examination revealed a typical presentation of right inferotemporal BRVO with a wedge-shaped distribution of intraretinal haemorrhages (Figure 1), while optical coherence tomography (OCT) demonstrated significant macular edema (Figure 2). Her intraocular pressure was found to be within the normal range.

Subsequently, she was treated initially with three intravitreal injections of aflibercept 40 mg/ml (Eylea®, Bayer, Leverkusen, Germany) followed by a further five injections administered as per clinical response over the subsequent 12 months. She was investigated for general risk factors for BRVO; a general screening was carried out in primary care including a battery of routine blood tests. Blood glucose, lipids, cholesterol, triglycerides and blood pressure were all found within the normal range. The patient was a non-smoker with no pre-existing health conditions; she denied any history of systemic disease. There was no relevant family history.

Interestingly, during the course of her ocular treatment, she developed acute dyspnea and was admitted under the respiratory team, eight months following initial presentation with BRVO. The patient was initially treated with broad-spectrum intravenous antibiotics and intravenous fluids for suspected infective pneumonia. As several courses of intravenous antibiotics did not resolve her symptoms, she was investigated with a thorax CT, which showed evidence of ILD. The patient was reviewed by the rheumatology team; she denied any rheumatological symptoms except for Raynaud’s phenomenon and had no obvious features of myositis. Nonetheless, a thorough investigation of myositis-specific autoantibodies (MSA) including the ASS autoantibody panel was conducted. The patient tested positive for anti-PL7 antibodies and was thus diagnosed with ASS. She was treated acutely with corticosteroids followed by cyclophosphamide and mesna intravenous infusions, which were received on a pro re nata basis as an outpatient.

Regarding her other further investigations as an inpatient, the myeloma screen was reported normal. However, alanine aminotransferase level was raised at 90 u/L, and C-reactive protein was found elevated at 34 mg/L.

Historically, ASS was not considered a separate entity and was in fact regarded as a subset of DM or PM depending on their clinical presentation. However, in this case, the patient was reviewed by the rheumatology team and found to have little features of DM, but was described to have Raynaud’s phenomenon with no particular signs of scleroderma. Although the prevalence of ILD (86%) is more common than that of myositis (76%) in ASS, myositis usually presents earlier than ILD [10]. Occasionally, the symptoms of ILD such as dyspnoea can be the first presenting symptom, with an estimated 33% of ASS patients presenting with ILD preceding myositis [5].

The diagnosis of PM/DM would usually be done by electromyography (EMG) and is associated with elevated muscle enzymes serum creatinine kinase and aldolase levels, which were normal in this clinically amyopathic patient. As ASS may present without myositis, it is important to be aware of the diagnostic criteria (Table 1; adapted from Witt et al., 2016) [2].

**Table 1:** ASS diagnostic criteria (adapted from Witt et al. 2016)* *[2] ASS: antisynthetase syndrome; tRNA: transfer ribonucleic acid

Connors et al. (2010)	Solomon et al. (2011)	
Required: presence of anti-aminoacyl tRNA synthetase antibody	Required: presence of anti-aminoacyl tRNA synthetase antibody	
PLUS one or more of the following clinical features: Raynaud’s phenomenon, arthritis, interstitial lung disease, fever (not attributable to another cause), mechanic’s hands (thickened and cracked skin on hands, particularly at fingertips)	PLUS two major/one major and two minor criteria:	
	Major	Minor
	Interstitial lung disease (not attributable to another cause), polymyositis or dermatomyositis as per Bohan and Peter criteria	Arthritis, Raynaud’s phenomenon, mechanic’s hands

Regarding treatment for her ophthalmic condition, she was managed with a course of three monthly anti-vascular endothelial growth factor (anti-VEGF) intravitreal aflibercept 40 mg/ml injections. This was followed by a further course of bimonthly injections over 12 months, to maintain dry macula and excellent visual acuity. Visual acuity improved gradually during the course of aflibercept injections, with binocular vision restored to 6/6 within one year. During a review six months after the last injection, the patient was noted to have a stable vision in both eyes.

Systemically, corticosteroids and immunosuppressants are the pillars of ASS management; however, a subset of patients will be refractory to steroids. Our patient continues to be under rheumatology follow-up for ASS monitoring.

## Discussion

Retinal vein occlusion (RVO) is the second most common retinal vascular disorder, with BRVO being the most common type of RVO having a global incidence of 0.44% [11]. BRVO is diagnosed mostly by clinical examination in conjunction with OCT and fluorescein angiography (FA). Management comprises laser photocoagulation, anti-VEGF intravitreal injections, steroids (for macular oedema), as well as optimising the management of risk factors in partnership with primary care. Hypertension, hyperlipidemia, ocular hypertension, and glaucoma are well-established risk factors of BRVO when adjusted for age, but associations have also been made with thrombophilic disorders: hyperhomocysteinemia and anti-cardiolipin antibodies have been identified thus far.

Systemic autoimmune diseases have been known to manifest as vaso-occlusive retinopathies, and various pathophysiology mechanisms have been described including vasculitis, vasospasm, immune-complex deposition and hypercoagulability. Zöller et al. have described autoimmune diseases as hypercoagulable disorders, which is attributed to their inflammatory role in cytokine cascade-mediated upregulation of pro-coagulants, inhibition of the protein C system and inhibition of fibrinolysis and the development of autoantibodies [12]. Three cases of the extra-ocular vaso-occlusive disease in ASS have been reported in the literature: a case of vaso-occlusive digital ischaemia in a 61-year-old Caucasian lady with anti-PL7 antibodies [13]; a case of pulmonary vaso-occlusive disease in a 55-year-old male with anti-Jo-1 antibodies [14]; and a case of a concurrent stroke at the time of ASS diagnosis in a 52-year-old Southeast Asian lady with anti-Jo-1 antibodies [15]. Interestingly, the patient in the first case developed vaso-occlusive disease prior to ILD symptoms, similar to our case, indicating that vaso-occlusive disease may be a precursor to severe ILD in patients with anti-PL7 antibodies.

Numerous studies have reported retinal vessel occlusion in multiple systemic autoimmune diseases, secondary to retinal vasculitis as well as secondary vasospasm. Wang et al. have reported a case of central retinal vein occlusion (CRVO) in a 25-year-old woman with DM. They reported evidence of vitreous cells in the affected eye and hence proposed that inflammation of the central retinal vein due to DM was the mechanism leading to the CRVO [16]. Two cases of retinal vasculitis secondary to ASS have also been published. The first was a case of a 50-year-old lady with anti-Jo-1 antibodies who developed ocular symptoms five days after hospitalisation for non-resolving pneumonia, fever, myositis and rash on hands and feet [7]. More recently, Donovan et al. described another case of a young 31-year-old woman with established ASS with ILD presenting with retinal vasculitis with significant macular thickening and vascular leakage with peripheral non-perfusion in the affected eye [9].

ASS is known to have an acute phase response in its initial stage, and it is likely that smaller retinal vessels may be occluded first while ILD and myositis remain subclinical, which may explain the development of BRVO in our patient several months prior to ILD symptoms [5]. This atypical ASS case has highlighted the variable presentation of ASS and the importance of considering ASS in amyopathic patients with retinal vaso-occlusive disease as this may herald ILD. This patient demonstrated excellent final visual outcome with aflibercept injections and immunosuppressive therapy. As ASAb levels are known to correlate with disease activity [2], prompt diagnosis and management may reduce the risk of further retinal vaso-occlusion by reducing cytokine-mediated inflammation of vessels.

## Conclusions

Though rare, it is important to consider that ASS may have a systemic association with vaso-occlusive retinopathies. The common risk factors of ASS are age greater than 50 years and female gender. ILD may be the first or only symptom of ASS and occasionally retinal vaso-occlusive disease may prelude respiratory symptoms and therefore such patients should be investigated for ASAbs. Prompt diagnosis and treatment of BRVO and the initiation of immunosuppressive therapy in these patients can lead to excellent final visual outcomes.
